# On the Physiological Position of Fibrin

**Published:** 1859-07

**Authors:** Levin S. Joynes

**Affiliations:** Professor of Institutes of Medicine in the Medical College of Virginia


### EXTRACTS.

### ON THE PHYSIOLOGICAL POSITION OF FIBRIN.

BY LEVIN S. JOYNES, M.D., PROFESSOR OF INSTITUTES OF MEDICINE IN THE MEDICAL COLLEGE OF VIRGINIA.

[From the *Virginia Medical Journal.*]

We can hardly do justice to this very able article in the brief extracts our space permits us to make from it, and yet it contains too much of interest to allow us to pass it by unnoticed.

Prof. Joynes gives a very just statement of the theory held by some late physiologists, viz: "that fibrin, so far from being a peculiarly organizable or plastic material, and the immediate pabulum of the most highly vitalized tissues, is, in reality, an *excrementitious compound*, not at all available for nutrition, and to be reckoned among those elements which have arisen in the blood from its own decay, or have reverted to it from the waste of the tissues, and are in process of elimination from the system."

Prof. Joynes adheres to the older theory, until recently held by all physiologists, which considers fibrin to be a most important element for the nutrition, formation and repair of the tissues; and sustains his opinion by the following arguments:

1*st*. "Fibrin is a constituent of the chyle. Evident indications of it are found in the fluid drawn from lacteals of an animal in full digestion, at their very issue from the intestine; but its quantity progressively increases by the transformation of albumen, as the chyle moves along the vessels towards the thoracic duct, and through it into the venous system; and a further increase takes place as the blood passes from the venous to the arterial side of the circulation. We may affirm, therefore, that the *proportion of fibrin increases as the products of digestion approach the points where materials are needed for the nutrition of tissue;* and we may ask if fibrin be an excrementitious product, why should it appear *in the chyle* directly after its absorption? We cannot account for its presence here by the waste of tissue, nor can we reasonably suppose the occurrence of a "retrograde metamorphosis". a destructive change in the products of digestion as soon as they are absorbed.

*2nd.* "Fibrin is normally found only in the *nutritive fluids* of the economy—the blood, chyle and lymph. It is not a constituent of any *excretion*, as are all those constituents of the blood which are admitted to be excrementitious,—such as carbonic acid, urea, uric acid, creatine, etc.

*3d.* "Fibrin is nature's agent for the *arrest of hemorrhage.* When vessels are divided, the coagulation of the blood is the means by which their occlusion is mainly effected, and the flow permanently arrested. If the blood contained no fibrin, and were therefore not coagulable, hemorrhage, even from the lightest wound, could never be arrested by the efforts of nature. But for the same protective property every separation of a gangrenous part would be attended with bleeding. Effusion of fibrin is also the means by which suppuration is circumscribed, and prevented from assuming that diffuse character which is sometimes so destructive. In these several particulars, fibrin performs offices which are singularly *conservative.* Can we say as much for any of these products of wear and tear which constitute the true offal of the system? It has been aptly remarked that the organism *bears an increase of fibrin better than a diminution.* Witness the comparative gravity of sthenic inflammation and the severer grades of typhoid fever. Not so with any organic compound of the excrementitous class. The accumulation of these in the blood is the signal of urgent peril.

*4th.* "Is it mere fancy that sees in the *spontaneous coagulation* of fibrin, and the definite position which its particles usually assume in solidifying, the indication of a special tendency to organization? And is it unwarrantable to argue therefrom the possession of a certain degree of *vitality?* All attempts to ascribe this coagulation to the operation of mere chemical or physical influences have failed. It is a change which fibrin always undergoes of its own accord, when not kept *moving in contact* with living parts, whatever be the external condition in other respects. Whenever, in the course of the circulation, the plasma of the blood is effused from the capillaries in the midst of the tissues for their nutrition, the fibrin being now *at rest,* is free to pass into the solid state, and enter into combination with the tissue or tissues of which it is the appropriate food. We have no just ground for affirming that fibrin is the only immediate tissue-forming ingredient of the blood; that albumen, for example, which abounds there, must pass through the form of fibrin before combining with any living structure. The probabilities are all against such an exclusive view. But that fibrin is a *specially and eminently organizable or histogenetic* material, this, I am convinced, is a truth which cannot be successfully controverted."

The whole article will abundantly repay perusal.

